# Proenkephalin and the risk of new‐onset heart failure: data from prevention of renal and vascular end‐stage disease

**DOI:** 10.1002/clc.23729

**Published:** 2021-10-30

**Authors:** Johanna E. Emmens, Jozine M. ter Maaten, Frank P. Brouwers, Lyanne M. Kieneker, Kevin Damman, Oliver Hartmann, Janin Schulte, Stephan J. L. Bakker, Rudolf A. de Boer, Adriaan A. Voors

**Affiliations:** ^1^ Department of Cardiology University of Groningen, University Medical Center Groningen Groningen The Netherlands; ^2^ Department of Cardiology Haga Teaching Hospital The Hague The Netherlands; ^3^ Department of Internal Medicine University of Groningen, University Medical Center Groningen Groningen The Netherlands; ^4^ SphingoTec GmbH Hennigsdorf Germany

**Keywords:** enkephalins, glomerular filtration rate, heart failure, NT‐proBNP, proenkephalin

## Abstract

**Background:**

Enkephalins of the opioid system exert several cardiorenal effects. Proenkephalin (PENK), a stable surrogate, is associated with heart failure (HF) development after myocardial infarction and worse cardiorenal function and prognosis in patients with HF. The association between plasma PENK concentrations and new‐onset HF in the general population remains to be established.

**Hypothesis:**

We hypothesized that plasma PENK concentrations are associated with new‐onset HF in the general population.

**Methods:**

We included 6677 participants from the prevention of renal and vascular end‐stage disease study and investigated determinants of PENK concentrations and their association with new‐onset HF (both reduced [HFrEF] and preserved ejection fraction [HFpEF]).

**Results:**

Median PENK concentrations were 52.7 (45.1–61.9) pmol/L. Higher PENK concentrations were associated with poorer renal function and higher NT‐proBNP concentrations. The main determinants of higher PENK concentrations were lower estimated glomerular filtration rate (eGFR), lower urinary creatinine excretion, and lower body mass index (all *p* < .001). After a median 8.3 (7.8–8.8) years follow‐up, 221 participants developed HF; 127 HFrEF and 94 HFpEF. PENK concentrations were higher in subjects who developed HF compared with those who did not, 56.2 (45.2–67.6) versus 52.7 (45.1–61.6) pmol/L, respectively (*p* = .003). In competing‐risk analyses, higher PENK concentrations were associated with higher risk of new‐onset HF (hazard ratio [HR] = 2.09[1.47–2.97], *p* < .001), including both HFrEF (HR = 2.31[1.48–3.61], *p* < .001) and HFpEF (HR = 1.74[1.02–2.96], *p* = .042). These associations were, however, lost after adjustment for eGFR.

**Conclusions:**

In the general population, higher PENK concentrations were associated with lower eGFR and higher NT‐proBNP concentrations. Higher PENK concentrations were not independently associated with new‐onset HFrEF and HFpEF and mainly confounded by eGFR.

## 1 INTRODUCTION

Enkephalins are endogenous opioid peptides that exert cardiodepressive effects, such as reducing heart rate and inhibiting norepinephrine release, as well as improving renal function by increasing renal blood flow and urinary output.^1^, ^2^, ^3^, ^4^, ^5^ Proenkephalin (PENK) is a stable surrogate for enkephalins.^3^ In subjects from the general population, higher concentrations of PENK were associated with a higher risk of development of chronic kidney disease (CKD).^6^, ^7^ In patients with an acute myocardial infarction, higher plasma PENK concentrations have been associated with an increased risk of development of heart failure (HF).^8^ In patients with established HF, PENK concentrations were elevated and higher concentrations have been associated with HF severity, worse(ning) of renal function (reflected by both glomerular and tubular renal markers), and adverse clinical events.^9^, ^10^, ^11^, ^12^ It remains to be established whether higher concentrations of PENK are also associated with an increased risk of new‐onset HF. We, therefore, investigated the association between higher PENK concentrations and new‐onset HF in the general population.

## 2 METHODS

### 2.1 Patient population

**FIGURE 1:**
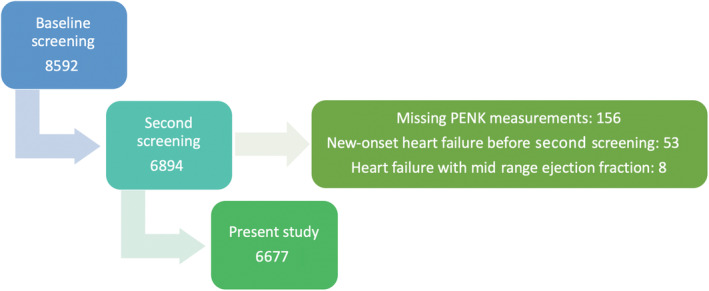
Flow diagram of in‐ and exclusion of patients. PENK, proenkephalin

The prevention of renal and vascular end‐stage disease (PREVEND) study was designed to prospectively investigate the natural course of urinary albumin excretion (UAE) and its association with the development of cardiorenal disease in the general population.^13^ From 1997 to 1998, all inhabitants of Groningen (The Netherlands) aged 28–75 years were asked to complete a questionnaire and send a vial containing early morning urine. Among respondents, 6000 subjects with a morning UAE ≥10 mg/L and 2592 randomly chosen subjects with UAE <10 mg/L were included. These 8592 subjects (4291 men, 4301 women) comprised the cohort that participated in the baseline screening assessment (1997–1998). From 2001 to 2003, the second screening followed (*n* = 6894), which was the starting point of the present study. Among these subjects, those who had already developed HF before the second screening assessment or were classified as having HF with midrange ejection fraction (left ventricular ejection fraction [LVEF] 41%–49%) were excluded (*n* = 53 and *n* = 8, respectively), as well as subjects with missing PENK values (*n* = 156), resulting in a study population of 6677 subjects (Figure 1).

The PREVEND study was approved by the medical ethics committee of the University Medical Center Groningen and was conducted in accordance with the Declaration of Helsinki. Written informed consent was obtained from all participants.

### 2.2 Data collection and measurements

All participants completed a self‐administered questionnaire regarding demographics, cardiovascular and renal disease history, smoking habits, alcohol consumption, and medication use prior to the baseline screening assessment. Medication use was verified with community pharmacies. Blood pressure was measured on the right arm, every minute for 10 and 8 minutes, respectively during two examination visits of the second screening visit. The mean of the last two recordings from each of the two examinations was used. Fasting blood samples were obtained in the morning and stored at −80°C. All participants collected two consecutive 24‐hour urinary specimens, subsequently stored at −20°C.

PENK was measured in plasma using a sandwich immunoassay targeting PENK amino acids 119–159 (SphingoTec GmbH, Hennigsdorf, Germany) as described previously.^14^ The lower detection limit of the assay was 7 pmol/L and mean interassay coefficients of variation was 5.7% in the measuring range 10.9–686.3 pmol/L. Total cholesterol, high‐density lipoprotein cholesterol, and low‐density cholesterol were determined as previously described.^15^ Serum creatinine measurement was performed by an isotope dilution mass spectrometry traceable enzymatic method (Roche Diagnostics, Mannheim, Germany). UAE was measured by nephelometry with a threshold of 2.3 mg/L and intra‐ and interassay coefficients of variation of 2.2% and 2.6%, respectively (Dade Behring Diagnostic, Marburg, Germany). N‐terminal pro brain natriuretic peptide (NT‐proBNP) and high‐sensitivity C‐reactive protein were measured as previously described.^16^, ^17^

### 2.3 Definitions

Estimated glomerular filtration rate (eGFR) was calculated using the CKD‐EPI creatinine formula.^18^ PENK was investigated according to varying degrees of glomerular function and glomerular damage defined by Kidney Disease: Improving Global Outcomes (KDIGO) GFR and albuminuria categories.^19^ KDIGO GFR and albuminuria categories “high risk” and “very high risk” versus “low risk,” or “moderately increased risk” were used to investigate interactions between presence/severity of kidney disease and PENK concentrations with regards to outcomes. Type 2 diabetes was defined as a fasting glucose of ≥7.0 mmol/L, a non‐fasting glucose of ≥11.1 mmol/L, or the use of antidiabetic medication. Left ventricular hypertrophy was defined according to the Cornell criteria on electrocardiography: a value of >2440 mm/ms as resulting from RaVL+SV_3_ (with 6 mm added in women) multiplied by QRS duration.

### 2.4 New‐onset heart failure

Details on the methodology for identifying new‐onset HF in PREVEND have been published previously.^20^ In brief, hospital records from both hospitals in Groningen, the University Medical Center Groningen and Martini Hospital, were checked for the presence of HF at baseline and for new‐onset HF. This was done by recording signs, symptoms, and objective evidence of HF. Permission to access hospital records was granted by the local Ethics Committees. Criteria were used in accordance with the European Society of Cardiology Heart Failure Guidelines applicable at the time.^21^ Each case was validated anonymously by two different HF experts including clinical charts, hospitalization, and physician office records of suspected cases. LVEF at time of diagnosis was used to define HF with reduced ejection fraction (HFrEF) and HF with preserved ejection fraction (HFpEF; LVEF ≤40% or ≥50%, respectively).

### 2.5 Cardiac and cardiovascular events and mortality

Cardiovascular endpoints were obtained through the Dutch national registry of hospital discharge diagnoses (PRISMANT), and adjusted according to detection in hospital records. Cardiac events (which were classified as being fatal or nonfatal) included acute myocardial infarction (ICD‐10 code 410), acute and subacute ischaemic heart disease (411), coronary artery bypass grafting, and percutaneous coronary angioplasty. Cardiovascular events (also classified as being fatal or non‐fatal) included cardiac events with the addition of stroke (subarachnoid hemorrhage [430], intracerebral hemorrhage [431], other intracranial hemorrhage [432], or occlusion or stenosis of the pre‐cerebral [433], or cerebral arteries [434]), and vascular interventions. Data on mortality was obtained from Statistics Netherlands to allow for competing risks analysis.^22^

### 2.6 Follow‐up

Time to events was defined from the date of the subject's second screening visit until the date of first new‐onset HF, cardiovascular events, death, or January 1, 2011. If a person had moved to an unknown destination, the date of last contact served as the censor date.

### 2.7 Statistical analysis

Based on the population size and range of PENK, PENK was divided into quintiles. Data are presented as mean ± SD when normally distributed, as median (Q1–Q3) for skewed variables, and as frequency (percentage) for categorical variables. Trends over PENK quintiles were statistically tested with the Cochran–Armitage trend test, Jonckheere–Terpstra test, or a linear regression model for categorical, skewed, or normally distributed variables, respectively. Otherwise, continuous normally distributed variables were tested with the student independent *t*‐test or analysis of variance (ANOVA), skewed variables with the Mann–Whitney *U* or Kruskal–Wallis test, and categorical variables with *χ* ^2^ tests.

Determinants of PENK concentrations were analyzed using univariable and multivariable regression analyses, in which all variables with *p* < .1 in univariable analysis were included in the multivariable analysis and subjected to the backward elimination method. For all linear regression analyses, the assumption of linearity and normal distribution of residuals was checked, as well as checks for outliers. If necessary, variables were transformed using natural logarithm, including PENK. Variables in multivariable regression models were checked for multicollinearity, which led to exclusion of age from the model due to multicollinearity with eGFR, with weak contributory value from age. Variables with *p* < .05 were retained in the final multivariable regression model. Competing‐risk regression analysis was used to assess whether PENK concentrations were associated with new‐onset HF, HFrEF, and HFpEF, where death was considered a competing risk in all analyses. In analyses pertaining HFrEF and HFpEF specifically, the other HF entity was additionally considered a competing risk. Competing‐risk regression analysis was executed using the cmprsk package, which uses Fine–Gray regression. Competing‐risk regression models were adjusted for sex, eGFR, and body mass index (BMI), and results are expressed as hazard ratios (HRs) per doubling of PENK with their corresponding 95% confidence intervals (CIs). The assumption of proportionality of hazards and linearity were checked in all analyses. In addition, interactions were evaluated in cox proportional hazard models between PENK concentrations and sex, KDIGO risk categories, and presence of eGFR <60 ml/min/1.73 m^2^. Cox proportional hazard models were also constructed for cardiovascular events to evaluate the prognostic predictability of log doubling of PENK concentrations, adjusted for sex and eGFR. Additional packages that were used in the analysis included the packages clinfun, DescTools, foreign, Hmisc, ggplot2, ggpmisc, lm.beta, nephro, psych, survival, and survminer. A two‐tailed *p*‐value <.05 was considered statistically significant. All statistical analyses were executed using R (version 3.4.3, R Foundation for Statistical Computing, Vienna, Austria).

## 3 RESULTS

### 3.1 Baseline characteristics according to plasma PENK concentrations

In the current study cohort, the mean age was 54 ± 12 years, and 3360 (50.3%) of subjects were female. Median plasma PENK concentrations were 52.7 (45.1–61.9) pmol/L in the overall study cohort, and 54.9 (47.2–64.0) pmol/L and 50.7 (43.3–59.3) pmol/L in women and men, respectively (*p* < .001). Subjects with higher PENK concentrations were, among others, older, more often female, had a lower BMI, were more often on antihypertensive treatment, had a lower eGFR, and had higher concentrations of NT‐proBNP, serum creatinine, and urea (Table 1; all *p* for trend <.001). UAE approximated a U‐shape over quintiles of PENK (*p* < .001). In Table S1, PENK concentrations are represented over KDIGO GFR and albuminuria categories, showing increasing PENK concentrations over GFR categories in all albuminuria categories (all *p* < .001), and also an increase of PENK concentrations over albuminuria categories in all GFR categories except G3b (all *p* < .05).

**TABLE 1:** Baseline characteristics of the PREVEND study in relation to quintiles of PENK concentrations

Variables	PENK (pmol/L)					*p* for trend
	Q1, *n* = 1336 39.2 (35.9–41.6) Min 19.1, max 43.4	Q2, *n* = 1335 46.7 (45.1–48.2) Min 43.4, max 49.7	Q3, *n* = 1335 52.7 (51.2–54.4) Min 49.8, max 56.0	Q4, *n* = 1335 59.7 (57.8–61.8) Min 56.0, max 64.4	Q5, *n* = 1336 71.7 (67.7–78.9) Min 64.4, max 532.3	
Clinical characteristics						
Age (years)	52 ± 11	53 ± 11	53 ± 12	53 ± 12	57 ± 14	**<.001**
Sex (female), *n* (%)	498 (37)	623 (47)	671 (50)	755 (56)	813 (61)	**<.001**
Race (Caucasian), *n* (%)	1278 (96)	1267 (95)	1278 (96)	1276 (96)	1258 (94)	.172
BMI (kg/m^2^)	28.0 ± 4.6	27.0 ± 4.2	26.5 ± 4.1	26.1 ± 4.2	26.0 ± 4.3	**<.001**
Systolic blood pressure (mm Hg)	128 ± 17	127 ± 18	125 ± 18	124 ± 20	128 ± 21	**<.001**
Diastolic blood pressure (mm Hg)	75 ± 9	74 ± 9	73 ± 9	72 ± 9	73 ± 9	**<.001**
Heart rate (bpm)	69 ± 10	69 ± 10	68 ± 10	68 ± 10	69 ± 10	.097
LVH according to Cornell, *n* (%)	26 (2)	22 (2)	29 (2)	26 (2)	33 (2)	.270
Medical history, *n* (%)						
Coronary heart disease	45 (3)	27 (2)	37 (3)	44 (3)	79 (6)	**<.001**
Diabetes mellitus	129 (10)	69 (5)	58 (4)	63 (5)	68 (5)	**<.001**
Smoking or quit **≤**1 year	472 (35)	512 (38)	515 (39)	506 (38)	518 (39)	.075
Medication, *n* (%)						
Lipid‐lowering treatment	124 (9)	110 (8)	105 (8)	138 (10)	166 (12)	**<.001**
Antidiabetic treatment	59 (4)	42 (3)	40 (3)	35 (3)	42 (3)	**.047**
Antihypertensive treatment	271 (20)	257 (19)	226 (17)	262 (20)	414 (31)	**<.001**
Laboratory values						
Hemoglobin (g/dl)	8.6 ± 0.8	8.6 ± 0.8	8.5 ± 0.7	8.4 ± 0.8	8.4 ± 0.8	**<.001**
Sodium (mmol/L)	141 (139–142)	141 (140–142)	141 (140–142)	141 (140–142)	141 (140–142)	**<.001**
Potassium (mmol/L)	4.2 (4.0–4.4)	4.2 (4.1–4.4)	4.2 (4.0–4.4)	4.2 (4.1–4.4)	4.2 (4.1–4.4)	**<.001**
NT‐proBNP (pg/ml)	31 (16–62)	36 (17–70)	41 (22–73)	45 (22–85)	58 (30–115)	**<.001**
Glucose (mmol/L)	5.0 (4.5–5.5)	4.8 (4.5–5.3)	4.7 (4.4–5.2)	4.7 (4.4–5.2)	4.8 (4.4–5.3)	**<.001**
ASAT (U/L)	23 (20–28)	22 (19–26)	22 (19–26)	22 (19–26)	22 (19–26)	**<.001**
ALAT (U/L)	19 (14–28)	18 (13–25)	17 (13–24)	16 (12–22)	16 (12–21)	**<.001**
Total cholesterol (mmol/L)	5.4 (4.7–6.1)	5.4 (4.8–6.1)	5.4 (4.7–6.1)	5.4 (4.7–6.1)	5.4 (4.7–6.2)	.956
HDL cholesterol (mmol/L)	1.1 (1.0–1.4)	1.2 (1.0–1.4)	1.2 (1.0–1.4)	1.2 (1.1–1.5)	1.3 (1.1–1.5)	**<.001**
LDL cholesterol (mmol/L)	3.3 (2.7–4.1)	3.5 (2.7–4.2)	3.6 (2.9–4.2)	3.3 (2.7–4.0)	3.4 (3.0–4.2)	.843
CRP (mg/L)	1.5 (0.6–3.3)	1.4 (0.7–3.1)	1.3 (0.6–3.1)	1.3 (0.6–2.9)	1.4 (0.7–3.1)	.095
Kidney function						
Creatinine (μmol/L)	70 (61–78)	69 (61–78)	71 (62–80)	71 (63–81)	75 (66–87)	**<.001**
eGFR (ml/min/1.73 m^2^)	98 ± 12	96 ± 13	94 ± 13	92 ± 15	84 ± 19	**<.001**
Urea (mmol/L)	5.0 (4.2–5.8)	5.0 (4.2–5.9)	5.0 (4.3–5.9)	5.1 (4.3–6.0)	5.4 (4.5–6.4)	**<.001**
Urinary albumin excretion (mg/24 h)	9.4 (6.7–17.0)	9.1 (6.3–16.6)	8.4 (6.0–14.9)	7.9 (5.9–14.1)	8.9 (5.8–20.4)	**<.001**
Urinary creatinine excretion (mg/24 h)	1541 (1279–1825)	1408 (1171–1703)	1352 (1142–1665)	1298 (1084–1553)	1206 (1009–1460)	**<.001**
Urinary urea excretion (mmol/24u)	246 (182–333)	230 (172–302)	226 (169–301)	222 (167–293)	208 (156–270)	**<.001**

Abbreviations: ALAT, alanine transaminase; ASAT, aspartate aminotransferase; BMI, body mass index; CKD, chronic kidney disease; CRP, C‐reactive protein; eGFR, estimated glomerular filtration rate; HDL, high‐density lipoprotein; LDL, low‐density lipoprotein; LVH, left ventricular hypertrophy; NT‐proBNP, N terminal pro brain natriuretic peptide; PENK, proenkephalin.

### 3.2 Main correlates of PENK concentrations

**FIGURE 2:**
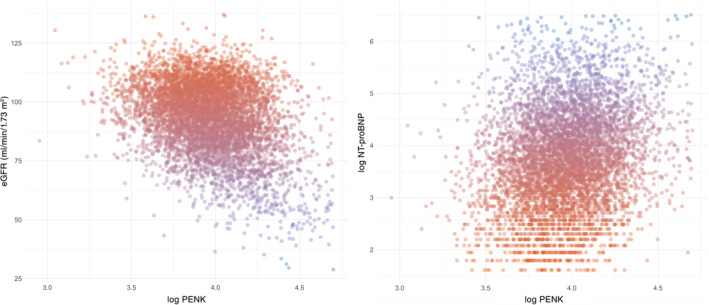
Correlation plots of PENK with eGFR and NT‐proBNP. Spearman correlation coefficients: eGFR, −0.276 (*p* < .001); NT‐proBNP, 0.192 (*p* < .001). eGFR, estimated glomerular filtration rate; NT‐proBNP, N‐terminal pro brain natriuretic peptide; PENK, proenkephalin

Correlation plots showing the association between PENK concentrations and eGFR and NT‐proBNP, respectively, are displayed in Figure 2. The strongest independent correlates of higher log‐transformed PENK were lower eGFR, lower log urinary creatinine excretion, and lower BMI (all *p* < .001; Table 2). The adjusted *R* ^2^ of the model was 0.276.

**TABLE 2:** Multivariable linear regression analysis for PENK^a^

Variable	Standardized beta	*T*	*p*‐value
eGFR	−0.379	−22.922	**<.001**
Urinary creatinine^a^	−0.173	−11.186	**<.001**
BMI	−0.130	−8.795	**<.001**
Smoking or quit **≤**1 year	0.091	6.511	**<.001**
Glucose^a^	−0.093	−6.416	**<.001**
Potassium^a^	0.058	4.204	**<.001**
Sodium^a^	0.056	4.119	**<.001**
Diastolic blood pressure	−0.054	−3.505	**<.001**
Urea^a^	0.052	3.380	**<.001**
Hemoglobin	−0.051	−3.308	**<.001**
Urinary albumin excretion^a^	0.045	3.012	**.003**
NT‐proBNP^a^	0.047	3.007	**.003**
Antihypertensive treatment	0.038	2.434	**.015**

*Note*: Adjusted *R* ^2^ of model: 0.276.

Abbreviations: BMI, body mass index; eGFR, estimated glomerular filtration rate; NT‐proBNP, N terminal pro brain natriuretic peptide; PENK, proenkephalin.

^a^ log‐transformed.

### 3.3 Plasma PENK concentrations are only univariably associated with new‐onset heart failure

In the current study cohort, a total of 221 subjects developed HF after a median follow‐up time (from the second screening visit) of 8.3 (7.8–8.8 years). The median time to HF diagnosis was 5.1 (2.9–6.7) years. In subjects who developed HF, median PENK concentrations were 56.2 (45.2–67.6) pmol/L and 52.7 (45.1–61.6) pmol/L in subjects who did not develop HF (*p* = .003). Among new‐onset HF cases, 127 subjects developed HFrEF, and 94 subjects HFpEF. In univariable competing‐risk regression analysis (Table 3), PENK concentrations were significantly associated with a higher risk of new‐onset HF (HR = 2.09 [95% CI 1.47–2.97] per doubling of PENK, *p* < .001), new‐onset HFrEF (HR = 2.31 [95% CI 1.48–3.61] per doubling of PENK, *p* < .001), and new‐onset HFpEF (HR = 1.74 [95% CI 1.02–2.96] per doubling of PENK, *p* = .042). After adjustment for sex and its main determinant eGFR, PENK concentrations were no longer associated with new‐onset HF and HFrEF. After additional adjustment for BMI, PENK concentrations were also no longer associated with new‐onset HFpEF. There was no interaction between plasma PENK concentrations and sex, KDIGO risk category, nor with presence of eGFR <60 ml/min/1.73 m^2^ at baseline with respect to all three outcomes.

**TABLE 3:** Competing‐risk regression analysis for PENK^a^ predicting new‐onset heart failure, also stratified per HFrEF and HFpEF

Outcomes	Univariable		Adjusted for sex and eGFR		Additionally adjusted for BMI	
	HR (95% CI)	*p*‐value	HR (95% CI)	*p*‐value	HR (95% CI)	*p*‐value
HF (*n* = 221)	2.09 (1.47–2.97)	**<.001**	0.85 (0.60–1.20)	.360	1.07 (0.75–1.53)	.720
HFrEF (*n* = 127)^b^	2.31 (1.48–3.61)	**<.001**	1.09 (0.71–1.68)	.690	1.26 (0.80–1.96)	.320
HFpEF (*n* = 94)^c^	1.74 (1.02–2.96)	**.042**	0.59 (0.35–0.99)	**.044**	0.80 (0.47–1.36)	.400

Abbreviations: BMI, body mass index; CI, confidence interval; HF, heart failure; HFpEF, heart failure with preserved ejection fraction; HFrEF, heart failure with reduced ejection fraction; HR, hazard ratio; PENK, proenkephalin.

^a^ log base 2 transformed.

^b^ In addition to death, HFpEF development was also considered a competing risk.

^c^ In addition to death, HFrEF development was also considered a competing risk.

**FIGURE 3:**
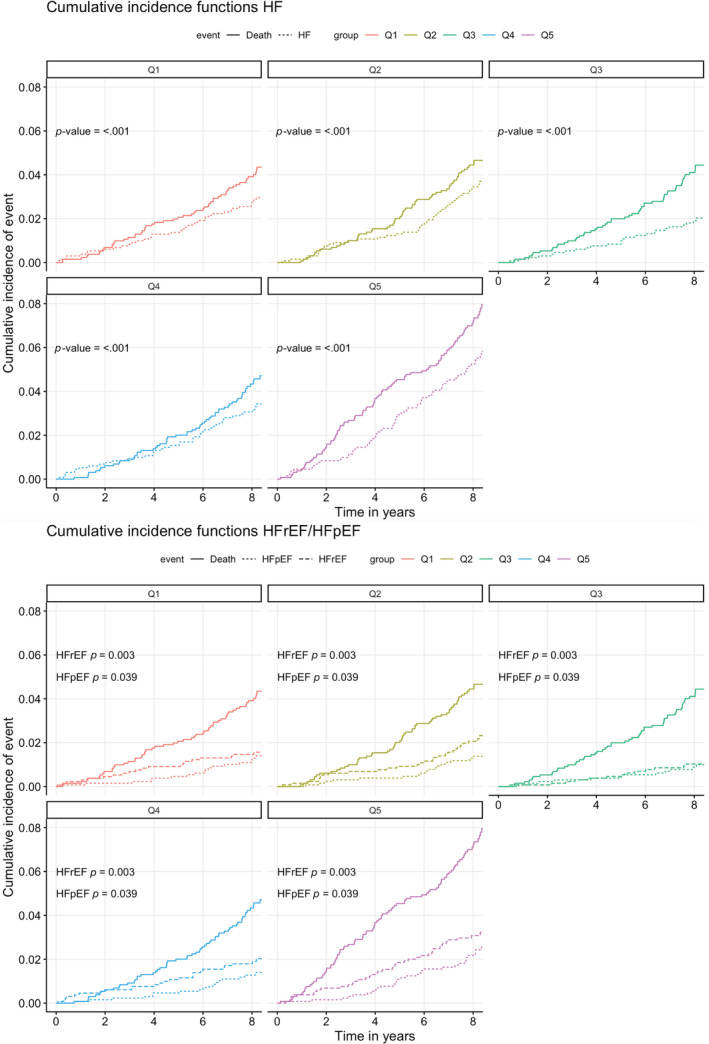
Competing risks cumulative incidence curves for new‐onset heart failure for quintiles of PENK concentrations. Cumulative incidence curves for new‐onset heart failure, heart failure with reduced ejection fraction, and heart failure with preserved ejection fraction stratified over quintiles of PENK concentrations. The indicators Q1 to Q5 represent the first quintile of PENK concentrations to the fifth quintile of PENK concentrations, respectively. HF, heart failure; HFpEF, heart failure with preserved ejection fraction; HFrEF, heart failure with reduced ejection fraction; PENK, proenkephalin

Competing risks cumulative incidence curves for new‐onset HF, HFrEF, and HFpEF respectively over quintiles of PENK concentrations illustrate an increasing risk with higher quintiles of PENK concentrations (Figure 3; *p* < .001 for HF; *p* = .003 for HFrEF; *p* = .039 for HFpEF).

In Table S2 the association of PENK with new‐onset HF, HFrEF, and HFpEF was analyzed per quintile of PENK concentrations. The frequency of new‐onset HF and HFrEF increased over ascending PENK quintiles (*p* = .003 and *p* = .019 respectively). The fifth and highest PENK quintile was univariably associated with new‐onset HF (HR = 1.92 [95% CI 1.29–2.84], *p* = .001) and new‐onset HFrEF (HR = 2.06 [95% CI 1.20–3.52], *p* = .009), but not after adjustment for sex and eGFR.

### 3.4 Plasma PENK concentrations and cardiac and cardiovascular events

Non‐fatal cardiac events, non‐fatal cardiovascular events, and fatal cardiovascular events occurred in 359, 434, and 38 subjects respectively. Plasma PENK concentrations were univariably associated with all three events (Table S3; HR = 1.50 [95% CI 1.14–1.98] per doubling of PENK, *p* = .004 for non‐fatal cardiac events; HR = 1.55 [95% CI 1.20–2.00] per doubling of PENK, *p* < .001 for non‐fatal cardiovascular events; and HR = 4.07 [95% CI 2.22–7.49] per doubling of PENK, *p* < .001 for fatal cardiovascular evens), but not after adjustment for sex and eGFR. There was no interaction present between plasma PENK concentrations and sex, KDIGO risk category, or presence of eGFR <60 ml/min/1.73 m^2^ at baseline.

## 4 DISCUSSION

In this study, we show data from the novel renal marker PENK in subjects from the general population. Those with higher plasma PENK concentrations were older, more often female, had lower eGFR, and higher concentrations of NT‐proBNP. The main independent correlates of higher PENK concentrations were lower eGFR, lower urinary creatinine excretion, and lower BMI. Higher PENK concentrations were univariably associated with new‐onset HF, HFrEF, and HFpEF in competing‐risk regression analysis, but this association was mainly confounded by low eGFR. The association of PENK concentrations was similarly attenuated by low eGFR with regards to other cardiovascular outcomes.

### 4.1 PENK in patients diagnosed with heart failure

Enkephalins of the endogenous opioid system have several cardiovascular effects, including reducing myocardial contractility, blood pressure, and heart rate, and renal effects including increasing renal blood flow and urinary output through delta‐opioid receptors which are highly expressed in kidney tissue.^1^, ^2^, ^23^ In addition, they inhibit sympathetic nervous system activation by inhibiting catecholamine release and sympathetic vascular constriction.^1^, ^24^ In a previous study in patients with HF, we observed that higher concentrations of PENK were associated with more severe heart failure, worse renal function, and increased mortality.^9^ We therefore hypothesized that PENK and the opioid system could be a common pathway affecting both the heart and the kidney, a so‐called “cardiorenal connector.” In this pathway, elevated PENK concentrations could either be detrimental, a counter‐regulatory response, or both protective and detrimental where at first the response is protective, but later becomes maladaptive.^2^, ^9^, ^25^ In other studies in patients with HF plasma PENK concentrations were also elevated and were associated with worse(ning) renal function, HF severity, and adverse clinical events.^10^, ^11^, ^26^ Due to the pronounced associations between PENK and HFpEF with renal dysfunction and CKD, PENK might be particularly important in HFpEF, where PENK concentrations have indeed been shown to be elevated and associated with indices of renal dysfunction, measures of diastolic dysfunction, and poor prognosis.^12^ In our previous study, higher PENK concentrations were associated with higher HFpEF prevalence.^9^

### 4.2 PENK in the general population

To our knowledge, the association between PENK concentrations and new‐onset HF in the general population has to date not been investigated at such a large scale including clear stratification of new‐onset HFrEF and HFpEF. One smaller study in 200 asymptomatic or minimally symptomatic community‐dwelling subjects (nearly all were men) showed that higher PENK concentrations were associated with a combined endpoint of death and HF.^27^ Median concentrations of PENK and associations were largely similar between this and our study. However, these patients were already selected based on the presence of conditions that increase the risk of developing HF or even already had structural heart disease (ACC/AHA Guidelines HF Stage A and B, respectively) and therefore differ from the general population of our study.

In the present study, the strong association between higher concentrations of PENK and renal dysfunction confirmed previous findings. PENK concentrations markedly increased over KDIGO GFR categories irrespective of albuminuria category and the main independent correlate of higher PENK concentrations was lower eGFR. The association between PENK concentrations and renal dysfunction might be explained by compensatory increased PENK production to exert kidney protective effects,^2^ or alternatively reflect impaired clearance since PENK is likely to be freely filtered through the glomerulus due to its low molecular weight (4586.60 g/mol) and is not known to have a binding protein.^5^ PENK has therefore also been suggested as a reflector of glomerular function especially in the acute setting.^5^, ^28^ Whatever the underlying mechanisms are, PENK concentrations have previously been associated with decline of eGFR and incident CKD in the general population,^6^ although in a previous study conducted in PREVEND this association was only found in men.^7^ The heart and the kidney are closely intertwined where failure of one can lead to failure of the other,^29^ which makes the relationship of PENK with renal dysfunction and CKD interesting to investigate with regards to new‐onset HF. We however did not observe an interaction between PENK concentrations and KDIGO risk category, nor with the presence of eGFR <60 ml/min/1.73 m^2^, with respect to new‐onset HF, but the numbers of subjects with a high‐risk category and/or eGFR <60 ml/min/1.73 m^2^ may have been too low to confidently show this interaction in these subpopulations of interest. PENK concentrations also showed an increase over albuminuria categories, even with normal eGFR or slightly/moderately decreased eGFR, implying that PENK concentrations might also be (more modestly) associated with glomerular damage. In multivariable regression analysis for PENK concentrations, UAE was retained in the final model, although strongly surpassed by eGFR. In a previous study conducted in PREVEND, no association was found between PENK concentrations and future CKD defined according to presence of albuminuria.^7^

We also found that higher PENK concentrations were associated with higher NT‐proBNP concentrations. This suggests that activation of the opioid system might be more pronounced in subjects with higher cardiac filling pressures. Alternatively, PENK might be a representation of poorer renal function meaning that higher NT‐proBNP concentrations are associated with poorer renal function. Although still well below lower reference limits, it has been shown that higher NT‐proBNP concentrations in the general population are associated with increased risk of all‐cause mortality and cardiovascular events.^30^ A relationship between the two biomarkers has previously been underscored by data indicating that opioid peptides may modulate natriuretic peptide release in HF, however, this has not been shown in healthy subjects.^31^

Importantly, the association of PENK concentrations with new‐onset HF and HFrEF was attenuated after adjustment for eGFR. The association between PENK concentrations and new‐onset HFpEF was attenuated after additional adjustment for BMI on top of eGFR. This suggests that PENK concentrations increase with declining eGFR, but that eGFR likely is the independent predictor of new‐onset HF, and not PENK by itself. In addition, subjects in our study population rarely reached the 99th percentile cutoff of >80 pmol/L (with a previously reported median (range) for PENK concentrations in the general population being 45 (9–518) pmol/L).^32^ Our results are in line with published data on several other newer biomarkers, for which it also has been shown that they have no or minimal incremental value with regards to the prediction of new‐onset HF.^33^ Clearly, the combination of a limited number of established risk factors, including age, sex, markers of renal dysfunction, and NT‐proBNP constitutes a firm base model, that can only be marginally supplemented by a few biomarkers.

Our findings are in contrast with a previous study showing that after myocardial infarction, higher concentrations of PENK were independently associated with a higher risk of developing HF.^8^ This discrepancy might be explained by previous studies showing that opioids play a role in the local regulation and response to cardiac injury where they offer cardioprotection through ischemic preconditioning.^2^ These data suggest that PENK is mainly expressed in response to cardiac injury to counteract its detrimental effects on the development of HF. In the general population, generally assuming there is not a significant extent of cardiac injury present, there might not be a reason yet for PENK to be expressed, as there are no detrimental effects to counteract. The same may hold true regarding the association between PENK concentrations and other cardiovascular endpoints.

### 4.3 Strengths and limitations

The findings of our study are based on a large, well‐characterized population of subjects from the general population and included a long follow‐up. HF diagnosis of both HFrEF and HFpEF was thoroughly validated and loss of follow‐up was minimal. Some rate of underdetection could however play a role especially regarding HFpEF, when diagnosis is not pursued from the general practitioner to the hospital. In addition, positioning of PENK in the general population could be performed quite extensively due to the large number of covariates that was available. Finally, optimal comparison of PENK values between different studies was ensured by the use of the same assay.

The fact that PENK concentrations were only measured at the second screening visit is a disadvantage of our study, as we could not study dynamic changes of PENK concentrations, notably in closer proximity to HF diagnosis. Furthermore, 61 patients already developed HF before the second screening visit and therefore had to be excluded. The results of our study are also predominantly based on subjects of Caucasian ethnicity, limiting the applicability of our results to other ethnicities. Lastly, the PREVEND cohort was enriched with subjects with increased albumin excretion, and although adjustments were applied for the presence or absence of albuminuria, we cannot exclude that it might have affected study results, however in pooled analyses with other cohort studies the results of the PREVEND studies always matched the overall results.^33^, ^34^

### 4.4 Conclusion

In subjects from the general population, higher plasma PENK concentrations were associated with lower eGFR and higher NT‐proBNP. Higher PENK concentrations were however not independently associated with new‐onset HFrEF and HFpEF and mainly confounded by eGFR. In the general population, PENK can be considered as a novel renal marker primarily related to renal glomerular function.

## CONFLICT OF INTEREST

The University Medical Center Groningen, which employs several authors, has received research grants and/or fees from AstraZeneca, Abbott, Bristol‐Myers Squibb, Novartis, Roche, Trevena, and ThermoFisher GmbH. Adriaan A. Voors received consultancy fees and/or research grants from: Amgen, AstraZeneca, Bayer, Boehringer Ingelheim, Cytokinetics, Merck, Myokardia, Novartis, Novonordisk, and Roche Diagnostics. Kevin Damman received consultancy fees from Boehringer Ingelheim and AstraZeneca. Oliver Hartmann and Janin Schulte are employed by SphingoTec GmbH, the manufacturer of the PENK immunoassay. Rudolf A. de Boer received speaker fees from Abbott, AstraZeneca, Novartis, and Roche. Johanna E. Emmens, Jozine M. ter Maaten, Frank P. Brouwers, Lyanne M. Kieneker, and Stephan J. L. Bakker have nothing to disclose.

## AUTHORS' CONTRIBUTIONS

Johanna E. Emmens, Jozine M. ter Maaten, and Adriaan A. Voors contributed to the conception/design of the work. Frank P. Brouwers, Lyanne M. Kieneker, Oliver Hartmann, Janin Schulte, Rudolf A. de Boer, and Stephan J. L. Bakker contributed to the acquisition of the data for the work. Johanna E. Emmens executed data analysis of the work. Jozine M. ter Maaten, Kevin Damman, and Stephan J. L. Bakker assisted in interpretation of data. Johanna E. Emmens drafted the manuscript. Johanna E. Emmens, Jozine M. ter Maaten, Frank P. Brouwers, Lyanne M. Kieneker, Oliver Hartmann, Janin Schulte, Rudolf A. de Boer, Adriaan A. Voors, Stephan J. L. Bakker, and Kevin Damman critically revised the manuscript for important intellectual content. All provided their approval for the final version of the manuscript.

## Supporting information


**Data S1.** Supporting information.

## Data Availability

The data that support the findings of this study are available from the corresponding author upon reasonable request.
