# Supplementary material for: Proenkephalin and the risk of new‐onset heart failure: data from prevention of renal and vascular end‐stage disease
Source: Clin Cardiol. 2021 Oct 30;44(12):1662–72. doi: 10.1002/clc.23729 (PMC8715404; doi:10.1002/clc.23729)
Supplement: Supplementary file 1 — Data S1. Supporting information. [file CLC-44-1662-s001.pdf]

Supplementary material

Proenkephalin and the risk of incident heart failure: data from PREVEND

Johanna E. Emmens[a], MD, Jozine M. ter Maaten[a], MD, PhD, Frank P. Brouwers[b], MD, PhD, Lyanne M. Kieneker[c], PhD, Kevin Damman[a], MD, PhD, Oliver Hartmann[d], PhD, Janin Schulte[d], PhD, Stephan J.L. Bakker[c], MD, PhD, Rudolf A. de Boer[a], MD, PhD, Adriaan A. Voors[a], MD, PhD

Affiliations

[a] University of Groningen, University Medical Center Groningen, Department of Cardiology, Groningen, the Netherlands

[b] Haga Teaching Hospital, Department of Cardiology, The Hague, the Netherlands

[c] University of Groningen, University Medical Center Groningen, Department of Internal Medicine, Groningen, the Netherlands

[d] Sphingotec GmbH, Hennigsdorf, Germany

Journal: Clinical Research in Cardiology

Corresponding author

Adriaan A. Voors

Department of Cardiology

University Medical Center Groningen

Hanzeplein 1, 9713 GZ, Groningen, The Netherlands

Tel: +31 (0)50 3616161

Fax: +31 (0)50 3618062

a.a.voors@umcg.nl

Supplementary Table 1. PENK concentrations according to KDIGO GFR and albuminuria categories

		Albuminuria categories (mg/gCr)			
		A1 (<30) N=5,827	A2 (30 – 300) N=597	A3 (>300) N=75	P for trend
eGFR categories (mL/min/1.73m^2)	G1 (≥ 90) N=3,896	50.6 (43.4 – 48.5)	48.3 (41.4 – 57.2)	51.3 (41.0 – 59.6)	0.040
	G2 (60 – 89) N=2,204	55.6 (48.1 – 64.7)	58.5 (48.7 – 68.3)	62.7 (54.1 – 77.4)	0.004
	G3a (45 – 59) N=166	71.9 (59.5 – 87.5)	77.2 (69.6 – 94.0)	86.6 (78.5 – 103.9)	0.003
	G3b (30 – 44) N=27	84.6 (72.8 – 102.0)	87.3 (77.7 – 131.8)	100.5 (81.7 – 119.4)	0.490
	G4/G5 (<30) N=12	110.3 (110.1 – 121.5)	186.9 (135.7 – 238.1)	188.5 (159.6 – 390.1)	0.039
	P for trend	<0.001	<0.001	<0.001	

(e)GFR, (estimated) glomerular filtration rate; KDIGO, Kidney Disease: Improving Global Outcomes; PENK, proenkephalin

Supplementary Table 2. Competing-risk regression for PENK* predicting incident heart failure, also stratified per HFrEF and HFpEF per quintile of proenkephalin

		Univariable		Adjusted for sex and eGFR		Additionally adjusted for BMI	
	N of events (%)	HR (95% CI)	P-value	HR (95% CI)	P-value	HR (95% CI)	P-value
Heart failure							
Log2 PENK	221 (3)	2.09 (1.47 – 2.97)	**<0.001**	0.85 (0.60 – 1.20)	0.360	1.07 (0.75 – 1.53)	0.720
Q1	38 (17)	1.0 (reference)	Ref	1.0 (reference)	Ref	1.0 (reference)	Ref
Q2	45 (20)	1.21 (0.78 – 1.86)	0.390	1.17 (0.74 – 1.84)	0.500	1.30 (0.83 – 2.05)	0.250
Q3	26 (12)	0.69 (0.42 – 1.14)	0.150	0.65 (0.38 – 1.08)	0.098	0.74 (0.44 – 1.25)	0.270
Q4	42 (19)	1.13 (0.73 – 1.75)	0.580	1.01 (0.63 – 1.61)	0.970	1.21 (0.76 – 1.95)	0.420
Q5	70 (32)	1.92 (1.29 – 2.84)	**0.001**	1.15 (0.73 – 1.82)	0.540	1.42 (0.89 – 2.27)	0.140
HFrEF							
Log2 PENK	127 (2)	2.31 (1.48 – 3.61)	**<0.001**	1.09 (0.71 – 1.68)	0.690	1.26 (0.80 – 1.96)	0.320
Q1	20 (16)	1.0 (reference)	Ref	1.0 (reference)	Ref	1.0 (reference)	Ref

**Q2**	28 (22)	1.42 (0.80 – 2.52)	0.230	1.69 (0.91 – 3.12)	0.096	1.80 (0.97 – 3.34)	0.064
**Q3**	14 (11)	0.71 (0.36 – 1.40)	0.320	0.81 (0.39 – 1.67)	0.560	0.88 (0.42 – 1.83)	0.730
**Q4**	25 (20)	1.27 (0.71 – 2.29)	0.420	1.41 (0.74 – 2.68)	0.290	1.58 (0.82 – 3.03)	0.170
**Q5**	40 (31)	2.06 (1.20 – 3.52)	**0.009**	1.57 (0.84 – 2.96)	0.160	1.80 (0.94 – 3.43)	0.075
HFpEF							
**Log2 PENK**	94 (1)	1.74 (1.02 – 2.96)	**0.042**	0.59 (0.35 – 0.99)	**0.044**	0.080 (0.47 – 1.36)	0.400
**Q1**	18 (19)	1.0 (reference)	Ref	1.0 (reference)	Ref	1.0 (reference)	Ref
**Q2**	17 (18)	0.97 (0.50 – 1.87)	0.920	0.72 (0.36 – 1.44)	0.350	0.84 (0.42 – 1.68)	0.630
**Q3**	12 (13)	0.68 (0.33 – 1.41)	0.300	0.51 (0.24 – 1.07)	0.075	0.62 (0.29 – 1.31)	0.210
**Q4**	17 (18)	0.97 (0.50 – 1.88)	0.930	0.68 (0.34 – 1.33)	0.260	0.87 (0.44 – 1.73)	0.680
**Q5**	30 (32)	1.73 (0.97 – 3.11)	0.065	0.79 (0.41 – 1.53)	0.480	1.04 (0.53 – 2.03)	0.920

CI, confidence interval; eGFR, estimated glomerular filtration rate; HFpEF, heart failure with preserved ejection fraction; HFrEF, heart failure with reduced ejection fraction; HR, hazard ratio; PENK, proenkephalin

Supplementary Table 3. Cox proportional hazards analysis for PENK* predicting cardiovascular outcomes

	Univariable		Adjusted for sex and eGFR	
Outcomes	HR (95% CI)	P-value	HR (95% CI)	P-value
Non-fatal cardiac events (n = 359)	1.50 (1.14 – 1.98)	0.004	0.88 (0.65 – 1.19)	0.404
Non-fatal cardiovascular events (n = 434)	1.55 (1.20 – 2.00)	<0.001	0.93 (0.70 – 1.22)	0.590
Fatal cardiovascular events (n = 38)	4.07 (2.22 – 7.49)	<0.001	0.93 (0.42 – 2.04)	0.857

*log base 2 transformed

CI, confidence interval; eGFR, estimated glomerular filtration rate; HR, hazard ratio; PENK, proenkephalin
